# Chasing after Non-cyanobacterial Nitrogen Fixation in Marine Pelagic Environments

**DOI:** 10.3389/fmicb.2017.01736

**Published:** 2017-09-08

**Authors:** Pia H. Moisander, Mar Benavides, Sophie Bonnet, Ilana Berman-Frank, Angelicque E. White, Lasse Riemann

**Affiliations:** ^1^Department of Biology, University of Massachusetts Dartmouth North Dartmouth, MA, United States; ^2^Marine Biology Section, Department of Biology, University of Copenhagen Helsingør, Denmark; ^3^Centre National de la Recherche Scientifique, IRD, Aix-Marseille Université, Université de Toulon Marseille, France; ^4^Mina and Everard Goodman Faculty of Life Sciences, Bar-Ilan University Ramat Gan, Israel; ^5^College of Earth, Ocean, and Atmospheric Sciences, Oregon State University Corvallis, OR, United States

**Keywords:** bacteria, diazotroph, DOM, mesopelagic, *nifH*, nitrogenase, oxygen minimum zone

## Abstract

Traditionally, cyanobacterial activity in oceanic photic layers was considered responsible for the marine pelagic dinitrogen (N_2_) fixation. Other potentially N_2_-fixing bacteria and archaea have also been detected in the pelagic water column, however, the activity and importance of these non-cyanobacterial diazotrophs (NCDs) remain poorly constrained. In this perspective we summarize the N_2_ fixation rates from recently published studies on photic and aphotic layers that have been attributed to NCD activity via parallel molecular measurements, and discuss the status, challenges, and data gaps in estimating non-cyanobacterial N_2_ fixation NCNF in the ocean. Rates attributed to NCNF have generally been near the detection limit thus far (<1 nmol N L^−1^ d^−1^). Yet, if considering the large volume of the dark ocean, even low rates of NCNF could make a significant contribution to the new nitrogen input to the ocean. The synthesis here shows that *nifH* transcription data for NCDs have been reported in only a few studies where N_2_ fixation rates were detected in the absence of diazotrophic cyanobacteria. In addition, high apparent diversity and regional variability in the NCDs complicate investigations of these communities. Future studies should focus on further investigating impacts of environmental drivers including oxygen, dissolved organic matter, and dissolved inorganic nitrogen on NCNF. Describing the ecology of NCDs and accurately measuring NCNF rates, are critical for a future evaluation of the contribution of NCNF to the marine nitrogen budget.

## Introduction

Biological dinitrogen (N_2_) fixation produces biologically available nitrogen (N) through reduction of atmospheric N_2_ to ammonium (${\text{NH}}_{4}^{+}$) (Postgate, 1998). In oligotrophic marine environments, N_2_ fixation can provide ~50% of the “new” N (Karl et al., 2002; Capone et al., 2005; Berthelot et al., 2017), and probably contributes more in hot spots such as the Western Tropical South Pacific (Bonnet et al., 2017). N_2_ fixation (diazotrophy) is catalyzed by the enzyme nitrogenase (Mortenson and Thorneley, 1979; Postgate, 1998). The *nifH* gene that encodes the dinitrogenase reductase is distributed in many phylogenetic groups of bacteria and archaea (Chien and Zinder, 1996), and used to assess the diversity and expression of the enzyme in marine diazotrophs (Zehr et al., 2003).

Cyanobacteria have traditionally been considered the most important diazotrophs in the ocean. The predominantly described taxa include the filamentous, bloom-forming *Trichodesmium*, heterocystous, symbiotic groups (e.g., diatom-diazotroph associations), and the unicellular cyanobacteria UCYN-A (*Candidatus* Atelocyanobacterium thalassa), UCYN-B (*Crocosphaera watsonii*), and UCYN-C (Zehr, 2011). Early molecular studies also reported the presence of non-cyanobacterial diazotrophs (NCDs) (Kirshtein et al., 1991; Zehr et al., 1995, 1998). These findings stimulated recent research on diversity, composition, and ecology of marine NCDs (Bombar et al., 2016), leading to a currently perceived emergence of a new paradigm: non-cyanobacterial N_2_ fixation (NCNF). Extensive research on free-living and symbiotic NCDs in soils has provided evidence of the activity, regulation, and the significance of these processes in terrestrial ecosystems (Postgate, 1998; Herridge et al., 2008; Hayat et al., 2010). While the diversity and abundance of marine NCDs indicate that NCNF could potentially have a large impact on the marine N budget, at present, the activity and contribution of marine NCNF to total N_2_ fixation remain poorly constrained.

In many oceanic waters, NCD sequences dominate the *nifH* gene pool (Riemann et al., 2010; Farnelid et al., 2011), and detection of transcripts of some of the NCD *nifH* genes suggests that at least some of the NCDs fix N_2_ in the oceanic water column and sediments (Bird et al., 2005; Church et al., 2005b; Halm et al., 2012; Brown and Jenkins, 2014; Moisander et al., 2014; Bentzon-Tilia et al., 2015b). However, gene expression cannot be equated with active N_2_ fixation rates without evidence of cell-specific activity. Current broadly available methods cannot discern the relative contribution of cyanobacteria and NCDs to measured N_2_ fixation rates. The contribution of NCNF could be studied by parallel rate measurements and molecular detection in areas where cyanobacterial diazotrophs are typically not present (such as the oceanic water column below the euphotic layer, i.e., aphotic waters). Here we summarize data from studies where N_2_ fixation rates were reported in parallel to molecular characterization of NCDs when cyanobacterial diazotrophs were *not* detected (presence and/or expression of the *nifH* gene; Table 1). Presumed NCNF rates ranged from undetectable to 8 and 0.89 nmol N L^−1^ d^−1^ in photic and aphotic studies, respectively (Table 1). The goal of this perspective is to synthesize emerging trends and data gaps from these studies and highlight future research directions.

**Table 1:** Compilation of photic and aphotic N_2_ fixation rates published with molecular data and associated with non-cyanobacterial diazotrophs in waters reportedly devoid of cyanobacterial diazotrophs.

**References**	**Location**	**Latitude**	**Longitude**	**Average rate or range of rates (nmol N L^−1^ d^−1^)**	**Depth (m)**	**Sequencing: Associated non-cyanobacterial phylotype genes and** transcripts	**qPCR: non-cyanobacterial diazotroph gene and** transcript abundance (*nifH* copies L^−1^)
SOUTH AND NORTH PACIFIC OCEAN							
Benavides et al., 2015	Western South Pacific	3–9°S	146–153°E	0.08–0.89	200–1000	Cluster I, III, IV, and *Trichodesmium* nd	0–900 nd
Halm et al., 2012	South Pacific Gyre	20–50°S	180°E–120°W	0.41.5	0–200	Cluster I, III nd	0–7^*^10^6^ $0–8*10^{4}$
Fernandez et al., 2011	Eastern Tropical South Pacific	1.5°N–17.8°S	86.9–70.8°W	0.01–3.5	70–400^a^	Cluster I, II, III nd	nd nd
Dekaezemacker et al., 2013	Eastern Tropical South Pacific	10–20°S	100–80°W	0–0.88	15–150	b b	b b
Turk-Kubo et al., 2014	Eastern Tropical South Pacific	10–20°S	100–80°W	b	10–200	Cluster I, III Cluster I	0–420 ~400
Loescher et al., 2014	Eastern Tropical South Pacific	2°N–16°S	85–74°W	0.4	0–350	Cluster I, III, IV nd	0–10^6^ 0–150
Bonnet et al., 2013	Eastern Tropical South Pacific	10–20°S	100–80°W	0–0.8	200–2,000	Cluster I, III nd	nd nd
Gradoville et al., 2017	Eastern Tropical South Pacific	20.1–26.3°S	104–77°W	0.5–5.1	5 m for N_2_ fix, 5–420 m for *nifH*	Cluster I, III nd	1.5 × 10^2^ −1.4 × 10^4^ nd
Hamersley et al., 2011	Southern California Bight	33.55°N	118.4°W	0.07	500–885	Cluster I, III, IV nd	nd nd
ATLANTIC OCEAN, ARCTIC OCEAN							
Sohm et al., 2011	South Atlantic Gyre and Benguela upwelling system	11–25°S	29°W–15°E	0.06–8	8 m for N_2_ fix, 8–110 m for *nifH*	nd nd	nd nd
Blais et al., 2012	Arctic Ocean coastal	69.3–75°N	69–134°W	0.02–4.45	5 m, DCM (30–57)	Cluster I, III nd	nd nd
INDIAN OCEAN							
Shiozaki et al., 2014	Indian Ocean	4°N–20°S	65–70°E	0.18–0.23	0–90	Cluster I nd	10^3^ nd
Jayakumar et al., 2012	Indian Ocean	13–19°N	64°–66°E	nd	110–175 (OMZ)	Cluster I, III, IV Cluster I, III	nd nd
MEDITERRANEAN SEA, RED SEA, AND BALTIC SEA							
Rahav et al., 2013a	Mediterranean Sea, Red Sea	29°55N–32°57N	34°29E–34°45E	0.01–0.38	150–720	Pseudomonas stutzeri-related nd	nd nd
Rahav et al., 2016	Eastern Mediterranean Sea	32°N	34°E	0.1–0.15	5	nd Cluster I: (Alpha−), and “other”	nd nd
Yogev et al., 2011	Eastern Mediterranean Sea	33.14–34.00°N	25°–33°E	0–0.3	0–160 m	Cluster I, II Cluster I, II	nd nd
Farnelid et al., 2013	Baltic Sea	57.20°N	20.03°E	0.44	200	Cluster I, II, III^d^ Cluster I, III	0–2^*^10^7^ $0–10^{4}$

*All rates are based on the ^15^N_2_ method and no conversions were made to originally reported values. The station location and depth information is specific for locations where **no** cyanobacterial diazotrophs were detected (unless otherwise indicated). Red letters indicate transcripts. Clusters I, II, III, and IV follow Chien and Zinder (1996)*.

a *Depths range from photic to aphotic*.

b *Dekaezemacker et al. (2013) reported rates and Turk-Kubo et al. (2014) reported corresponding molecular data*.

c *nd, data not available/not done*.

d *Nodularia transcripts were detected at abundances below the level of quantification via RT-qPCR and its transcripts were not detected by sequencing*.

## NCNF in photic waters

Most early studies of oceanic N_2_ fixation (Montoya et al., 1996; Karl et al., 2002; Hood et al., 2004; Mahaffey et al., 2005; Mulholland et al., 2006) and diazotroph diversity and activity (Zehr, 2011) focused on photic waters and cyanobacterial diazotrophs. Traditionally, the photic layer has been considered an optimal environment selecting for cyanobacterial N_2_ fixation, as abundant light energy (harnessed via photosynthesis) supplies the high energetic demands of the N_2_ fixation process, while dissolved inorganic nitrogen (DIN) limits non-diazotrophic autotrophs. Yet, since the first molecular studies (Zehr et al., 1998), *nifH* sequence libraries have revealed diverse NCD phylotypes from surface waters of various oceans and estuaries (Falcon et al., 2004; Church et al., 2005a; Langlois et al., 2005; Hewson et al., 2007; Moisander et al., 2008; Foster et al., 2009; Farnelid et al., 2011; Halm et al., 2012; Bentzon-Tilia et al., 2015b; Messer et al., 2016). Transcripts from a range of NCD groups have been recovered in surface layers (Man-Aharonovich et al., 2007; Halm et al., 2012; Loescher et al., 2014). One of the few consistent NCDs in photic layers is a gamma-proteobacterial cluster of sequences (termed γ-24774A11, Gamma A, or UMB; representatives of the same phylotype). Gamma A shows a wide distribution and expression of the *nifH* gene (Bird et al., 2005; Church et al., 2005b; Moisander et al., 2014; Langlois et al., 2015). This group appears to be broadly distributed across tropical and subtropical surface waters compared with cyanobacterial diazotrophs (Moisander et al., 2014; Bonnet et al., 2015; Langlois et al., 2015). Its presence exclusively in surface layers suggests either reliance on a photosynthetic machinery, rhodopsin, or photosynthesis products from other organisms. Once cultivated or its genome assembled, we can learn more about its autecology. A few other studies have detected N_2_ fixation from photic layers in the reported absence of cyanobacterial sequences (Yogev et al., 2011; Blais et al., 2012). However, due to the difficulty of demonstrating the absence of cyanobacterial diazotrophs, it has generally been difficult to prove that NCNF is active in photic layers.

The nitrogenase enzyme may be downregulated or inactivated by ${\text{NH}}_{4}^{+}$ (Zehr et al., 1997). As typically assumed for cyanobacterial diazotrophs (Zehr, 2011), DIN could negatively impact NCDs directly (physiological inhibition) or indirectly (NCDs outcompeted by faster growing non-diazotrophs). However, DIN availability may not always have immediate negative effects on NCDs. For example, Gamma A transcripts and Cluster III diazotrophs (Chien and Zinder, 1996) were found in the presence of micromolar concentrations of DIN in the upper layers of the Arabian Sea (Bird and Wyman, 2013). N_2_ fixation rates were also detected in the Benguela Upwelling System in the presence of micromolar nitrate and in the absence of cyanobacterial diazotrophs (Sohm et al., 2011). In other surface waters (Eastern Tropical South Pacific; ETSP), N_2_ fixation rates were similarly reported in the absence of cyanobacterial diazotrophs and presence of high DIN (Fernandez et al., 2011; Dekaezemacker et al., 2013; Gradoville et al., 2017). In Indian Ocean surface waters with a shallow nitracline, N_2_ fixation rates were reported in parallel with up to 10^4^ *nifH* gene copies L^−1^ of Gamma A, while cyanobacterial groups were undetectable (Shiozaki et al., 2014). Collectively these field studies suggests that NCDs may have a low sensitivity to DIN (Knapp, 2012), but recent work with cultivated NCDs suggest that responses to DIN may be strain specific (Bentzon-Tilia et al., 2015a).

PCR amplification biases likely influence our current data and conclusions of studies on NCDs. A recent study using metagenomic data from the TARA Oceans survey reports presence of nitrogenase containing Planctomycetes in oceanic photic waters and suggests they have a substantially greater abundance than the NCDs reported based on past PCR approaches (Delmont et al., 2017).

## NCNF in aphotic waters and oxygen deficient zones

Several studies have recently reported N_2_ fixation rates in the aphotic ocean where active autotrophic cyanobacterial diazotrophy is not expected. Early evidence for aphotic N_2_ fixation showed proteobacterial and archaeal diazotrophs and N_2_ fixation in hydrothermal vent fluids (Mehta and Baross, 2006). Deep-sea sediment archaea fixing N_2_ apparently function in associations with anaerobic methane oxidizers (Dekas et al., 2009, 2014). Proteobacterial *nifH* sequences were reported from the meso- to abyssopelagic water column in different oceans (Hewson et al., 2007; Moisander et al., 2008).

Laboratory studies have shown that nitrogenase activity in cells is reduced under increasing concentrations of oxygen, and the enzyme is irreversibly modified by oxygen *in vitro* (Fay, 1992; Berman-Frank et al., 2008). Thus, oxygen deficient zones (ODZs) that may span photic and aphotic layers could serve as sites of N_2_ fixation due to low oxygen, in parallel with N losses from these zones (Deutsch et al., 2007). The first studies reporting planktonic N_2_ fixation rates in aphotic waters were ones associated with ODZs in the Eastern Tropical North Pacific Ocean (ETNP) (Hamersley et al., 2011) and ETSP (Fernandez et al., 2011; Dekaezemacker et al., 2013). N_2_ fixation rates were reported in mesopelagic waters down to 400 and 800 m, and abyssopelagic waters down to 2,000 m (Fernandez et al., 2011; Bonnet et al., 2013; Loescher et al., 2014). These rates were attributed to various NCD DNA sequences, including Cluster I (Proteobacteria), II and III (Turk-Kubo et al., 2014). N_2_ fixation in the presence of NCD *nifH* expression was also reported in the aphotic ODZ of the Baltic Sea (Farnelid et al., 2013), and in the ODZ of the Arabian Sea (Jayakumar et al., 2012). These studies suggest that NCNF may be active in ODZs, however evidence of the relationship between oxygen and NCNF is variable. N_2_ fixation in the aphotic Western Tropical South Pacific had a negative correlation with dissolved oxygen (Benavides et al., 2015). However, mesopelagic N_2_ fixation rates in the Mediterranean Sea were reported from fully oxygenated waters (Rahav et al., 2013a), or were negatively correlated with apparent oxygen utilization values (i.e., N_2_ fixation was higher in more recently ventilated waters; Benavides et al., 2016). Gammaproteobacteria (Cluster I) and Cluster III generally dominated the *nifH* DNA sequences in these studies. In addition, both spatial coupling (Deutsch et al., 2007) and decoupling (Knapp et al., 2016; Bonnet et al., 2017) of N_2_ fixation and denitrification has been proposed. Collectively these studies suggest that NCNF is occurring in aphotic waters, and may correlate with oxygen availability, but the mechanisms by which oxygen and possibly other factors regulate NCNF in aphotic waters remain poorly known.

## The potential role of dissolved organic matter (DOM) in supporting NCNF

Organic particles may provide a site of low oxygen and a source of DOM that both can benefit specific bacterial attachment (Thiele et al., 2015; Dang and Lovell, 2016). If NCDs are heterotrophic (Riemann et al., 2010), overall DOM availability should play a role in controlling their growth and activity (Kirchman, 1990). Genomic and physiological analyses have confirmed that gamma- and alphaproteobacterial diazotrophs isolated from the Baltic Sea are heterotrophic and contain genes responsible for DOM metabolism (Bentzon-Tilia et al., 2015a). Evidence for DOM influence on NCDs was reported from the South Pacific Gyre (SPG) photic layers (Halm et al., 2012) and in the Mediterranean Sea (Rahav et al., 2016), where DOM originating from primary production was suggested to impact N_2_ fixation. Most of the SPG sequences were gammaproteobacterial, but represented different groups from those found in the ETSP (Fernandez et al., 2011; Turk-Kubo et al., 2014). Mesopelagic N_2_ fixation rates had a positive correlation with relatively labile DOM (Benavides et al., 2015), and were enhanced upon the addition of sugars, amino acids, or transparent exopolymeric particles (Bonnet et al., 2013; Rahav et al., 2013a; Loescher et al., 2014; Benavides et al., 2015).

In the eastern Mediterranean Sea, N_2_ fixation in the photic zone was uncoupled from primary production and correlated significantly and positively with bacterial production (Rahav et al., 2013b). However, even in coastal waters with high dissolved inorganic nutrient loads, organic carbon stimulated light and dark N_2_ fixation in a community containing cyanobacterial and NCD *nifH* phylotypes (Rahav et al., 2016). Saharan dust addition (serving potentially as a source of trace elements, nutrients, and/or DOM) enhanced N_2_ fixation and both NCD (Gamma A) and cyanobacterial diazotroph abundances in the North Atlantic (Langlois et al., 2012). Gamma A abundances were also enhanced by addition of sugars in the South Pacific (Moisander et al., 2012). Many cyanobacteria, including diazotrophs, take up organic forms of carbon (Hietanen et al., 2002; Church et al., 2004; Moisander et al., 2012; Benavides et al., 2017) thus rates of N_2_ fixation in mixed communities, measured after DOM amendments, may reflect responses of both cyanobacterial and NCDs. Whether some marine NCDs also use light as an energy source remains to be demonstrated (Bombar et al., 2016).

## Current and future challenges

The data synthesis here shows that while N_2_ fixation rates have been reported by several studies in waters dominated by NCD *nifH* sequences (Table 1), most studies of NCDs did not measure transcription. The *nifH* DNA sequences often do not appear as transcripts in the same samples, suggesting that some of the organisms are not active (Moisander et al., 2006; Short and Zehr, 2007; Yogev et al., 2011; Halm et al., 2012; Severin et al., 2015), thus it would be misleading to use *nifH* gene (DNA) data as proof for NCNF. Despite the common detection of cyanobacterial and NCD *nifH* in DNA sequence libraries and, at times, in transcripts, only a few studies from surface layers reported the *absence* of cyanobacterial diazotrophs when N_2_ fixation rates were detected (Table 1). Due to various methodological constraints, proving the absence of low numbers of cyanobacterial cells in a sample is difficult, if not impossible, yet such low abundance may be sufficient to result in detectable N_2_ fixation rates. Aphotic waters may be considered a good case study for NCNF, as photoautotrophic N_2_ fixation in these waters is conceivably absent. However, the common detection of diazotrophic cyanobacteria in aphotic layers (Hamersley et al., 2011; Farnelid et al., 2013; Benavides et al., 2015), possibly due to either settling material that could be viable upon experimental incubations (Agusti et al., 2015), or caused by contamination during sampling, complicate attributing measured aphotic N_2_ fixation rates to NCDs alone. Overall, the measured rates of *in situ* aphotic N_2_ fixation are higher than the parallel abundance and transcript numbers of NCDs would potentially support. Moreover, the reported cell-specific rates of NCDs are under debate (Turk-Kubo et al., 2014; Benavides et al., 2015; Bentzon-Tilia et al., 2015a; Gradoville et al., 2017).

Various factors of the ^15^N_2_ method, such as uncertainties in the ^15^N-labeling step, influence N_2_ fixation rate determination (Mohr et al., 2010; Grosskopf et al., 2012; Wilson et al., 2012). In addition, commercially available ^15^N_2_ gases are at times contaminated with substrates other than N_2_ (Dabundo et al., 2014), which could lead to false positive NCNF rates. An additional source of uncertainty that must be considered when assessing minimum quantifiable N_2_ fixation rates is the concentration and isotopic composition of particulate organic N (PON), which at typical concentrations requires large volumes in incubations (usually >4 L) to constrain rates in deep waters. While N_2_ fixation rates per volume are most informative for budgetary calculations, rates normalized to PON concentration would provide an additional measure for comparing rates across studies. Further, variability of the natural δ^15^N background of PON, changes in the δ^15^N of PON that may occur over the incubation period or due to substrate additions, and δ^15^N of the N_2_ pool, should be considered (Montoya et al., 1996; Gradoville et al., 2017). These sources of uncertainty are not routinely reported and detection limits are infrequently calculated. Taking into account these sources of error, Gradoville et al. (2017) estimated the minimum quantifiable N_2_ fixation rate in their study at ~0.4 nmol L^−1^ d^−1^ which is higher than many reported rates of NCNF (Table 1).

To our knowledge, direct field measurements of N_2_ fixation per cell are currently lacking for marine NCDs; such measurements would be important in assessing their contribution to N_2_ fixation rates, along with other efforts to separate signals of NCNF and cyanobacterial N_2_ fixation (see also Bombar et al., 2016). High NCD *nifH* diversity renders identification and quantification of biogeochemically significant individual groups challenging. The combination of *in situ* hybridization approaches using halogenated probes together with single-cell mass spectrometry (nanoscale secondary ion mass spectrometry; nanoSIMS) has recently advanced quantification of single-cell N_2_ fixation rates in UCYN-A (Thompson et al., 2012; Krupke et al., 2013). Similar approaches could provide insights into the NCNF in marine environments. Stable isotope probing and isotope microarrays could lead to valuable future insights (Seyler et al., 2014; Arandia-Gorostidi et al., 2017).

In describing the communities, sequencing depth and primer specificity influence what portion of the NCD community is detected. In addition, the detection limit for diazotroph transcripts or genes and the detection limit for N_2_ fixation are not necessarily equal. The quantification limit of quantitative PCR is often on the order of 10^2^ *nifH* gene copies L^−1^ (and often unreported), which could potentially result in false negatives for cyanobacterial diazotrophs, and subsequently, lead to false positive NCNF rates. Using small water volumes when abundance of cyanobacterial diazotrophs is low would increase the chances of reporting false negatives for these organisms. On the other hand, metabolic rate measurements of microbial samples brought to the surface from aphotic depths may be underestimated (Tamburini et al., 2013), making both rate and transcription analyses of deep communities challenging.

## Conclusions

Our compiled analysis of data illustrates that low N_2_ fixation rates were reported from marine environments where NCD abundance was high and cyanobacterial diazotrophs were low or undetected. NCD *nifH* sequences show a wider geographical and depth distribution in pelagic environments overall than their cyanobacterial counterparts. The emerging data suggest that the NCD communities are diverse but only a few groups have been identified that appear in several studies (Farnelid et al., 2011; Turk-Kubo et al., 2014). Studies on NCD *nifH* transcripts in aphotic layers are scarce or missing for many areas of the oceans.

We currently lack a fundamental understanding of the key players and environmental regulation of NCNF in the ocean. Ecophysiological data of the organisms contributing to these rates are still preliminary and incomplete. Moreover, the measured rates in most environments are generally “snap-shots” determined during a cruise/sampling foray and have not been examined over seasonal and annual cycles. In addition, several methodological concerns complicate interpretation of N_2_ fixation rate data. Modeling the significance of NCNF in marine N_2_ fixation remains a challenge due to these various constraints. If these rates are confirmed, however, NCNF could contribute significantly to new N inputs to the ocean. How factors such as temperature, DIN, oxygen, trace elements, and hydrostatic pressure drive the metabolic capacities and adaptations of NCNF are currently only partially revealed. The actual activities and taxon specific roles of marine NCDs remain enigmatic at present, as do their contributions to regional and global N_2_ fixation.

## Author contributions

PM and LR designed the study, wrote the initial drafts, and compiled the majority of Table 1. All authors wrote sections of the manuscript and contributed to Table 1.

### Conflict of interest statement

The authors declare that the research was conducted in the absence of any commercial or financial relationships that could be construed as a potential conflict of interest.
